# Supplementary figures and images for: Glycoprotein N-linked glycans play a critical role in arenavirus pathogenicity
Source: PLoS Pathog. 2021 Mar 1;17(3):e1009356. doi: 10.1371/journal.ppat.1009356 (PMC7951981; doi:10.1371/journal.ppat.1009356)

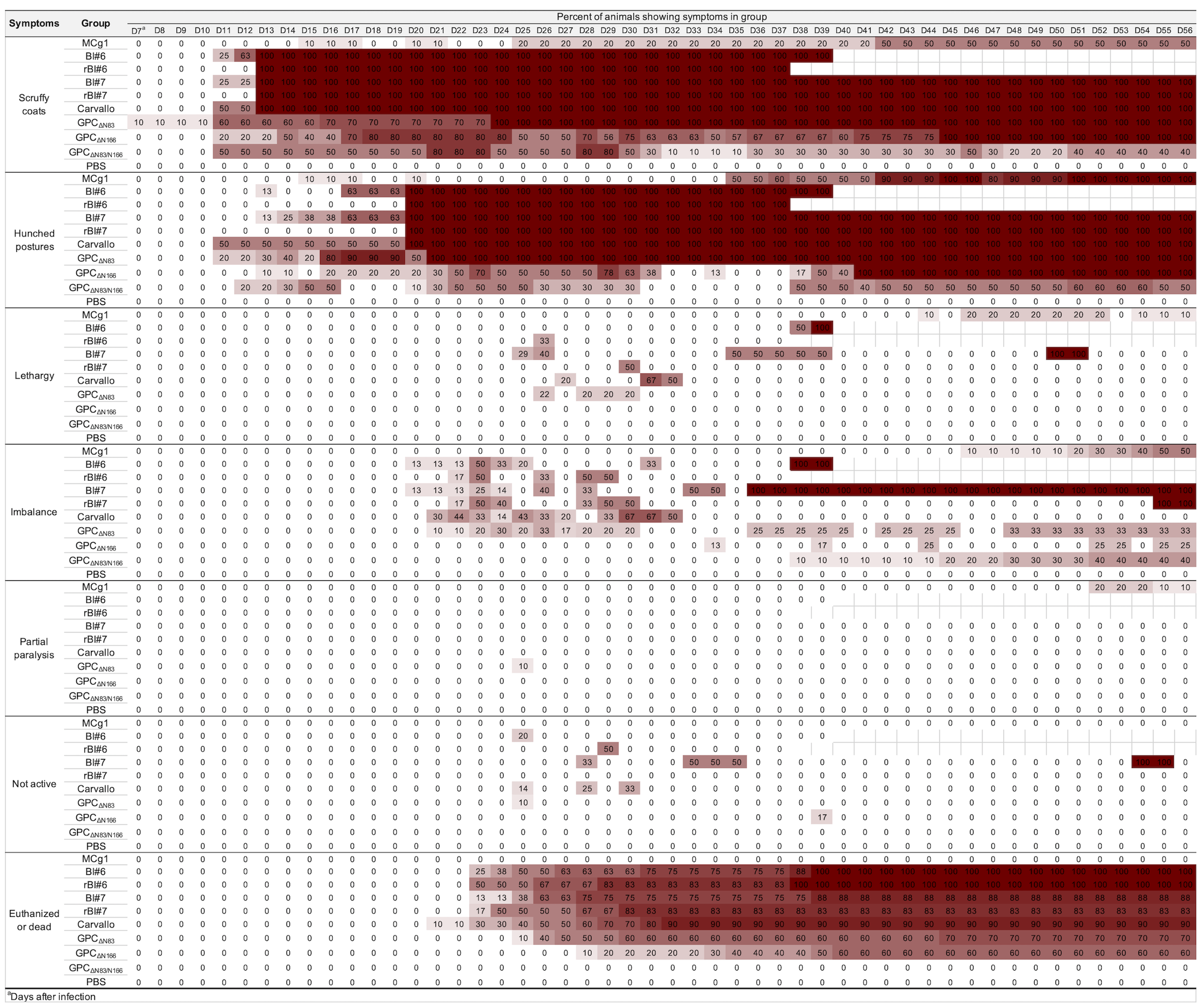

Supplement: S1 Fig — The heat map color and the indicated numbers represent the percentage of each clinical sign in animal groups. (TIF) [file ppat.1009356.s001.tif]

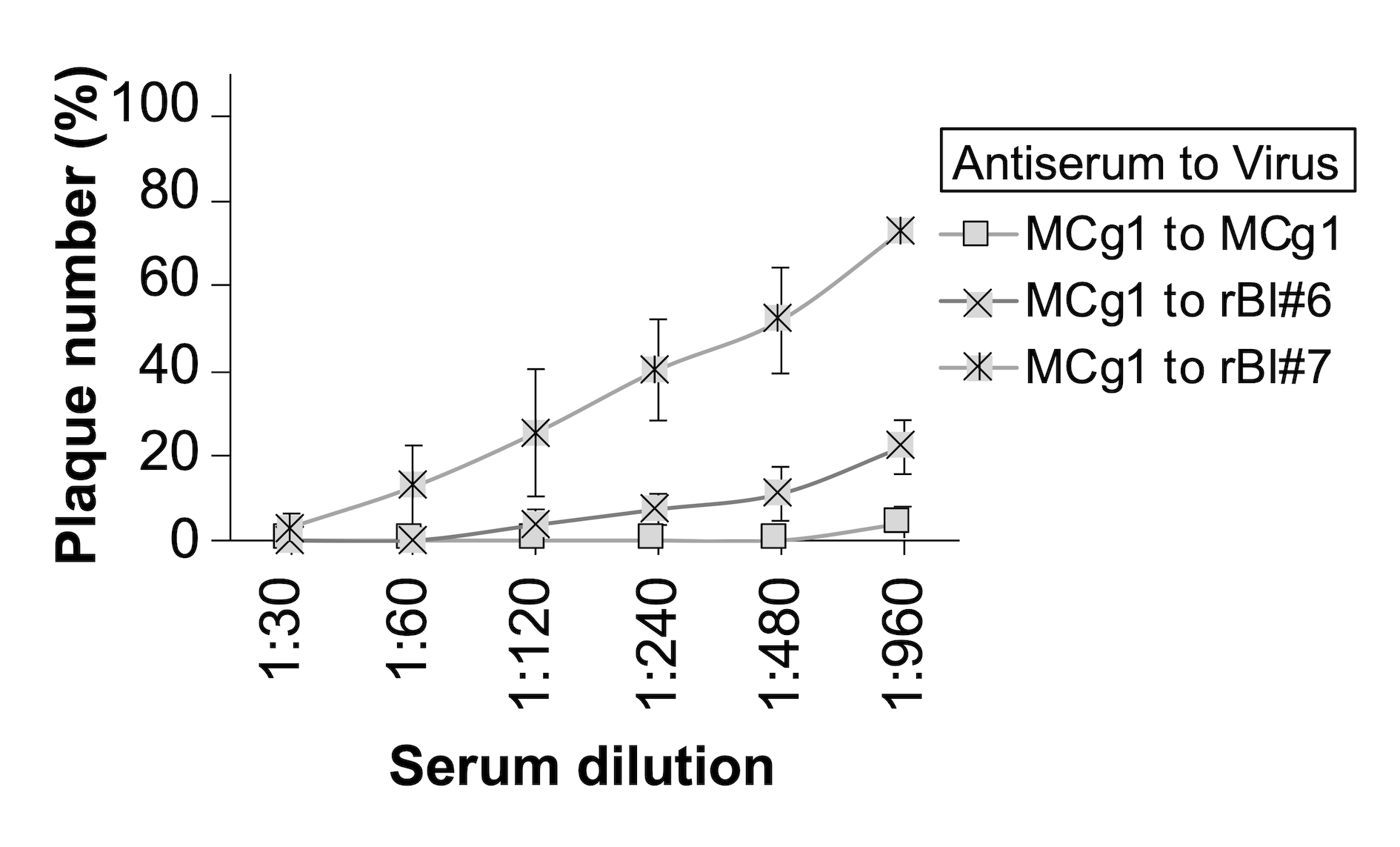

Supplement: S2 Fig — This graph represents the percentage of plaque numbers for each serum dilution. (TIF) [file ppat.1009356.s002.tif]
