# Supplementary material for: Glycoprotein N-linked glycans play a critical role in arenavirus pathogenicity
Source: PLoS Pathog. 2021 Mar 1;17(3):e1009356. doi: 10.1371/journal.ppat.1009356 (PMC7951981; doi:10.1371/journal.ppat.1009356)
Supplement: S1 Table — a No mutation was observed at the position. (DOCX) [file ppat.1009356.s003.docx]

| Table S1. Frequency of amino acid sequence substitution in GPC of MCg1 in animals. | | | | | | | | |
| --- | --- | --- | --- | --- | --- | --- | --- | --- |
| Position | | Substitution frequency for individual (n = 6-12) (Percentage) | | | | | | |
| GPC_85_ | GPC_168_ | #1  (33 dpi) | #2  (42 dpi) | #3  (33 dpi) | #4  (42 dpi) | #5  (33 dpi) | #6  (42 dpi) | #7  (42 dpi) |
| No mutation^a^ | No mutation | 100 | 22.2 | 0 | 62.5 | 0 | 0 | 0 |
| GPC_P85S_ | No mutation | 0 | 0 | 100 | 0 | 0 | 0 | 0 |
| No mutation | GPC_A168S/T_ | 0 | 55.6 | 0 | 25 | 25 | 27.3 | 80 |
| GPC_P85S_ | GPC_A168S/T_ | 0 | 22.2 | 0 | 12.5 | 75 | 72.7 | 20 |
| ^a^ No mutation was observed at the position. | | | | | | | | |
