# Supplementary material for: Glycoprotein N-linked glycans play a critical role in arenavirus pathogenicity
Source: PLoS Pathog. 2021 Mar 1;17(3):e1009356. doi: 10.1371/journal.ppat.1009356 (PMC7951981; doi:10.1371/journal.ppat.1009356)
Supplement: S2 Table — a”Yes” indicates the population contains the substitution at the position. b”No” indicates the population has a racesingle peak in the original sequence chromatograms. (DOCX) [file ppat.1009356.s004.docx]

| Table S2. Summary of amino acid changes in GPC of MCg1 in this study. | | | | | | | | | | | |
| --- | --- | --- | --- | --- | --- | --- | --- | --- | --- | --- | --- |
|  | Animal ID | | | | | | | | | | |
| Mutation | #2-1 | #2-2 | #2-3 | #2-4 | #2-5 | #2-6 | #2-7 | #2-8 | #2-9 | #2-10 |  |
| GPC_P85S_ | Yes^a^ | No^b^ | Yes | Yes | No | Yes | Yes | No | Yes | Yes |  |
| GPC_A168S/T_ | Yes | Yes | Yes | Yes | Yes | Yes | Yes | Yes | Yes | Yes |  |
| ^a^”Yes” indicates the population contains the substitution at the position. | | | | | | | | | | | |
| ^b^”No” indicates the population has single peak which is original sequence chromatograms. | | | | | | | | | | | |
