# Supplementary material for: Glycoprotein N-linked glycans play a critical role in arenavirus pathogenicity
Source: PLoS Pathog. 2021 Mar 1;17(3):e1009356. doi: 10.1371/journal.ppat.1009356 (PMC7951981; doi:10.1371/journal.ppat.1009356)
Supplement: Attachment — Submitted filename: PLOS Pathogens Response to reviewers Final.docx [file ppat.1009356.s005.docx]

**Response to Reviewers**

**Original Manuscript ID: PPATHOGENS-D-20-02227**

**Title: Glycoprotein N-linked glycans play a critical role in arenavirus pathogenicity**

We greatly appreciate the editors’/reviewers’ insightful and helpful suggestions and we have revised this submission in order to address all comments. Our responses are written in red.

Editor’s comments

1. The manuscript needs editing for English usage and grammar. Articles are missing or appear incorrectly in many instances, tenses are incorrect, as well as other issues (e.g. "reversed the T168A substitution on Cd#1 GP1" should be in).

Response: Thank you for the suggestion. The revised manuscript was proofread by a native English speaker.

2. p. 8, line 131. Fig. 1B does not show that the nucleotide substitutions were found in almost all infected animals. In fact, if I understand correctly, 3/4 animals maintained the MCg1 sequence. 4/7 is hardly all. I also didn't understand why those animals that had mutant viruses did not fall ill if these viruses were more pathogenic and resistant to neutralizing antibodies.

Response: As shown in Table S1, 3 out of 7 animals had the original MCg1 virus, but 2 of them also had mutant viruses. In other words, 6 out of 7 animals had mutant viruses. To clarify, we have revised from "in almost all infected animals (Fig 1B)." to **"** Our sequencing analysis identified the C341T and/or G590A/T nucleotide substitutions in the MCg1 S genome **(Fig 1B) in 6 out of 7 infected animals (Table S1). "** in lines 128-130 in resubmission.

It is true that MCg1 infected animals did not fall ill although more pathogenic mutant viruses emerged. We discussed this result in the revised manuscript in lines 406-416 as following:**“In MCg1-infected mice, although mutants with the glycosylation(s) at N83 and N166 emerged in the majority of animals, those mice did not succumb to infection like those infected by rBI#6 and rBI#7. It is likely that neutralizing antibodies elicited by MCg1 facilitated hosts to control mutant virus infections as a result of the race between neutralizing antibody production and the emergence of mutant viruses. In closely related Tacaribe virus infection, neutralizing antibodies was found to be produced at 8 dpi in IFN-αβ/γ R^-/-^ mice (****49****). In our study, mutants were not detected at 17 dpi. It is very likely that neutralizing antibodies were elicited before the emergence of mutant viruses in MCg1-infected animals. Although those mutants acquired additional glycans that rendered viruses less susceptible to neutralizing antibodies, data in Fig. 3 showed MCg1-infected mice developed antibody responses that could neutralize the mutants (rBI#6 and rBI#7) to some extent (>1:128). Thus, it is not unexpected that hosts survived despite the appearance of those mutants that otherwise are more virulent.”**

3. I found the morbidity curves a bit strange, because they go up and down (1E and 3E). Does this mean that some animals that showed signs of morbidity improved, and then later succumbed to disease.

Response: The editor is correct. The diseases in some animals improved transiently and worsened later as shown in Fig S1. The mechanism remains to be identified in future studies. One possibility is that infections were temporarily controlled by neutralizing antibodies in some cases but worsened once viruses were able to be disseminated into brains. We have added discussions in lines 416-420 as following: **“In some animals, especially those infected by MCg1, MACV GPC_ΔN166_ and MACV GPC_ΔN83/N166_, the diseases appeared to be alleviated or not overt temporarily and then exacerbated or appeared later (Fig S1). The mechanism remains to be investigated in future studies. One possibility is that infections were transiently controlled by antibodies and then worsened probably due to virus dissemination to the immune-privileged brain. ”**

Reviewer's Responses to Questions

**Part I - Summary**
Reviewer #1:  This study in general is well conducted and follows logical sequence.…

Response: I appreciate it.

Reviewer #2:  Overall, this is a well performed study with significant new findings that may contribute to the development of attenuated New World Arenaviral strains that could be used for vaccination.…

Response: I appreciate it.

Reviewer #3: The author have revisited an old finding in the biology of the arenaviruses. It has been known since the early days of studies of the cell biology of the group that glycosylation plays an important, even crucial role in the biosynthesis and expression of cogently folded GPC. From the work of *Wright* in the late 1980's who showed that glycosylattion of the GPC precursor was requires to express properly folded and immunoreactive GPC and to cleave the precursor to form the mature GP1/GP2 complex and for neutralizing antibody recognition. This was followed by the work of *Bonhomme* et al who showed in two papers published in 2011 and 2013 that glycosylation influenced both viral fitness and Tropism, and GP expression and function. Later work by *Sommerstein* and published in PLoS Pathogen demonstrated that the evasion from neutralizing antibody and virus persistence was promoted by a "glycan Sheld", and in a second paper, *Watanabe* et al showed that the Lassa Glycan Shield provides a model for Immunologic resistance. So the impact of the findings herein are quite predictable, and I have to ask whet is really new. It appears to this reviewer that this novel infornmation ois limited to the the non -conceptual information that is conveyed in the results, and certainly not to the concept that viral glycoprotein glycosylation is capable of an immunomodulating activity.

Response: We appreciate the reviewer’s constructive critiques. Accordingly, we have incorporated that literature and provided additional discussion in the resubmission as indicated below. As the reviewer pointed out, these important studies have collectively demonstrated that N-glycans are required for GPC expression, correct folding and cleavage. The glycans on LCMV GPC have also been found affecting virus tropism and fitness in cultured cells as well as masking important epitopes from antibody neutralization. In line with previous findings, our current study for the first time demonstrated the critical roles of specific GPC N-glycans in the pathogenicity of arenavirus *in vivo*. Importantly, we found that disruption of specific GPC glycosylation sites led to attenuation of a highly pathogenic arenavirus in animals without noticeable impact on the expression and cleavage of GPC or viral fitness in cultured cells. Those mutant viruses were more sensitive to antibody neutralization presumably due to loss of the glycans that mask important epitopes involved in virus entry. Interestingly, infection of animals with mutant viruses lacking specific GPC glycans elicited higher levels of neutralizing antibody to wild-type MACV, suggesting a role of these glycans on immunogenicity. These new findings may inform the development of vaccine candidates against pathogenic arenavirus infection.

Introduction section

- **“The ectodomain of arenavirus GPCs contains several N-linked glycosylation motifs that are important for the expression, proper folding and cleavage of GPC (****20-23****). For Old World lymphocytic choriomeningitis virus (LCMV), the addition or disruption of certain glycosylation sites on GPC is also shown to affect virus cell tropism and fitness in cultured cells (****24****). Furthermore, it has been reported that specific glycans on arenavirus GPCs mask important epitopes and thereby facilitate viral evasion of antiviral activities of neutralizing antibodies (****25-27****). However, the importance of GPC N-glycans in arenavirus pathogenicity has not been revealed. In this study, we report for the first time the critical role of specific N-glycans on GPC in the virulence of a hemorrhagic fever-causing arenavirus *in vivo*.“** in lines 74-82.
- “**Furthermore, we found that disruption of the specific glycosylation sites on MACV GPC led to attenuation of the highly pathogenic arenavirus in animals without noticeable influence on the expression and cleavage of GPC or viral fitness in cultured cells. Those mutant viruses were more sensitive to antibody neutralization presumably due to loss of the glycans that mask important epitopes involved in virus entry. Interestingly, infection of animals with MACV mutants lacking specific glycans on GPC elicited higher levels of neutralizing antibodies to wild-type MACV, suggesting these glycans have an impact on GPC immunogenicity. These new findings may inform the development of vaccine candidates against pathogenic arenavirus infections.** ” in lines 110-118.

Result section

- **“Our MCg1 infection data and previous studies show that the viruses often mutate to gain N-linked glycosylation sites (Fig 1)(****24****). To minimize the likelihood of reversion, we simultaneously mutated the first (N) and the third (S or T) amino acid residues in the N-linked glycosylation motif (N-X-S/T).”** in lines 234-237.

Discussion section

- **“No mutations occurred in MCg1 in cell culture experiments in our study. Nevertheless, a previous report showed that revertants appeared in serial passages of N-glycan deficient LCMV GPC_S116A_, LCMV GPC_T234A_ and LCMV GPC_S373A_ mutants (****24****) in cell cultures. To minimize the likelihood of genetic reversion, we disrupted the glycosylation site by changing 4-5 nucleotides to mutate both N and S/T of the N-X-S/T motif, instead of disrupting one amino acid with a single nucleotide substitution (Fig 8). No revertant was observed in the animals infected with the viruses for as long as 56 days. This approach may be useful in designing an attenuated arenavirus vaccine. “** in lines 325-332.
- **“N-glycans on GPC have been found to affect the tissue tropism of Old World LCMV(****25****). Further studies are required to understand how N-glycans on GPC alter the tissue tropism of New World arenaviruses.”** in lines 391-393.
- **“Based on this finding and the importance of the N-glycans of GPCs in proper folding and cleavage of GPCs (****20-22****,** **25****), wild-type MACV GPCs with glycans at N83/N166 are likely being folded properly and therefore are less likely be degraded and induce an immune response.”** in lines 397-400.

**Part II – Major Issues: Key Experiments Required for Acceptance**

Reviewer #1:

1. The authors associated increased virulence of the N83/N166 virus with its increased resistance to neutralization. Please discuss if this means the differences in the virulence between rBI#7 (or MACV) and MCg1 (or Candid#1) may not be revealed until humoral immunity is generated. Humans die in ~14 days after the onset of symptoms.

Response: We thank the reviewer for this comment. It is likely that the difference in virulence between the viruses with the two N-glycans (rBI#7 and MACV Carvallo) and without the two N-glycans (MCg1, MACV GPC_ΔN83/N166_ and Candid#1) is attributable to differences in the host's humoral immune response and virus sensitivity to neutralization. Accordingly, we have added the following discussion in lines 357-365 as following: **“The difference in virulence of the viruses with or without the two glycans could be explained by differences in the viral resistance to neutralization and humoral immune responsiveness. In our previous study of MACV Carvallo infection in animals, the neutralizing antibody titers to MACV were below the detection limit at 4 weeks of infection, when the majority of infected mice died (****29****). At the same time point, MCg1-infected animals developed neutralizing antibodies at titers of 1:42.4 (geometric mean, 33 dpi) against heterologous MACV Carvallo and 1:480 to >1:960 against homologous virus (see Table 2 of our previous publication ((****37****)). These results support the hypothesis that the two N-glycans on GPC not only render the virus refractory to neutralizing antibodies but also negatively affect the host humoral immune response.”**

2. The authors mentioned that the mutations restoring N166 glycan were more frequent than those restoring N83 glycan (lines 372-373) and that N166 glycan is more important for shielding neutralizing antibody epitopes (i.e. of the receptor-binding site) than the glycan at N83 (lines 273-274 and 294-295). Interestingly, while this phenomenon was observed in mice, mTfR1 is not believed an efficient receptor for MACV (PMC2268193). The authors can add this point in Discussion.

Response: Indeed, Radoshitzky et al. reported that MACV does not use mTfR1 as a receptor as efficiently as human TfR1 in their experiments. Accordingly, we have discussed this point in lines 342-347 as follows:**“Radoshitzky reported that MACV does not use murine TfR1 as a receptor as efficiently as human TfR1 in their experiments using pseudotype virus (****44****). Further studies may be needed to clarify the contribution of GPC glycans in the context of human TfR1. The crystal structure of MACV GP1 bound to human TfR1 (****39****) (Fig 6) support the idea that a higher importance of N166 glycan than that of N83 glycan is not limited to mice in MACV infection.”**

3. The authors also suggested increased proinflammatory cytokines, induced by increased degradation of the GPC, as a mechanism for lower virulence of the viruses lacking N166. Could higher abundance of GPC on the virion explain higher dissemination and virulence of the viruses containing N83/N166? This point should also be discussed.

Response: According to the reviewer’s comment, we have added the discussion in lines 394-405 as follows: “**Recently we have demonstrated that JUNV GPC lacking the N-glycosylation site at N166 accumulates in the form of dimers and trimers and is more readily degraded than the wild-type GPC within the lysosome (****33****). This phenomenon likely leads to increased antigen presentation and pro-inflammatory responses (****33****). Based on this finding and the importance of the N-glycans of GPCs in proper folding and cleavage of GPCs (****20-22****,** **25****), wild-type MACV GPCs with glycans at N83/N166 are likely being folded properly and therefore are less likely be degraded and induce an immune response. In addition, wild-type MACV GPC could be more efficiently incorporated into the particles, resulting in an increase in the density of the GP1/GP2 complex in the virions. A higher density of envelope glycoprotein in Ebola virus virions enhances the lectin receptors’ attachment and also enables a higher density of GP to escape from neutralizing antibodies (****48****). Thus, the density of the GP1/GP2 complex in virions should be investigated in the future to understand if it is influenced by N-glycosylations.”**

Reviewer #2: I have only one major comment:
Although neutralization is clearly an important function of antibodies, Fc-mediated effector functions may also play an important role in suppressing viremia. The removal of glycans can render the virus more sensitive to neutralization as the authors propose but it may also facilitate binding of antibodies in geometries that can better recruit effector functions. The authors could address this possibility by performing PRNT in the presence or absence of complement, for example. In addition, it will be worth discussing this possibility in the discussion.

Response: We appreciate the reviewer’s comment. We have added the discussion in lines 365-372: **“ In our recent study, we found that twenty non-neutralizing monoclonal antibodies against MACV GPC did not have an antibody-dependent cellular cytotoxicity (ADCC), and only neutralizing antibodies retained high ADCC reporter activity (****9****). This indicates that neutralizing antibodies against MACV Carvallo control the virus not only by binding to the receptor binding sites but also by ADCC in infected individuals. Furthermore, non-neutralizing antibodies have been reported to contribute to reducing LCMV clearance mediated *via* a complement-dependent manner (****45****). The removal of N-glycans may increase the sensitivity to antibodies *via* Fc-mediated effector functions, which should be investigated in future studies.”**

Reviewer #3: The paper is written with no historical context. With proper framing the findings of specific mutations and their effects on pathogenesis could be of interest. But to achieve that aim the manuscript needs a total re-write with the incorporation of a consideration of the known universe of findings. The potential novelty that the group could offer is the analysis of the high-containment pathogenesis of the various mutants. But this needs to be done according to state of the art standards with the incorporation of parameters of molecular pathogenesis and histopathology to differentiate the study from another run-of-the-mill pathology manuscript.

Response: As above mentioned, in accordance with the reviewer's comments, we have substantially rewritten the paper to take into account known findings and to clarify our new findings. Furthermore, we have modified the title from the role of glycans in “pathogenesis” to “**pathogenicity**”. We think the term “pathogenicity” is more appropriate to the study.

**Part III – Minor Issues: Editorial and Data Presentation Modifications**
Please use this section for editorial suggestions as well as relatively minor modifications of existing data that would enhance clarity.

Reviewer #1: Changing “Group” to “Plasma group” or “Serum group” will help to understand Tables 1 and 2. “Carvallo, 56 dpi” also needs an explanation.

Response: In accordance with the reviewer's comment, we have changed the name “Group” to **“Serum group”** and added an explanation for “Carvallo, 56 dpi”.

Reviewer #2: Minor issues:
1) The two PRNT tables are not sufficiently clear since the sera sources and the target viruses have the same names. Please clearly state the source of sera and the target virus in the PRNT.

Response: We have improved the presentation of the tables 1 and 2 to make them easier to understand and less misleading.
2) I couldn’t find a reference to Figure 7 in the text.

Response: We have referred to the figure (Assigned as Fig 8 in the revision.).
3) At some places the wrong references are being called (line 380 for example), please double check.

Response: Thank you for pointing this out. We have corrected the error.

4) I suggest to move figure S2B to the main text, as this figure shows some of the more significant results of this study.

Response: According to the reviewer’s suggestion, Fig S2 has been moved as Fig 3.

Reviewer #3: (No Response)
