# Supplementary material for: Glycoprotein N-linked glycans play a critical role in arenavirus pathogenicity
Source: PLoS Pathog. 2021 Mar 1;17(3):e1009356. doi: 10.1371/journal.ppat.1009356 (PMC7951981; doi:10.1371/journal.ppat.1009356)
Supplement: Attachment — Submitted filename: R2 PLOS Pathogens Response to reviewers Final.docx [file ppat.1009356.s006.docx]

**Response to Editor**

**Original Manuscript ID: PPATHOGENS-D-20-02227**

**Title: Glycoprotein N-linked glycans play a critical role in arenavirus pathogenicity**

We appreciate Editor's constructive comments. According to your comment, we have revised the sentence you pointed out as follows:

**“Since MACV and JUNV do not use murine TfR1 as a receptor (44-47), further studies are needed to clarify the contribution of GPC glycans in the context of human TfR1.”** in lines 342-344. We have cited the below papers in revising this sentence.

Ref 44.	Radoshitzky SR, Kuhn JH, Spiropoulou CF, Albarino CG, Nguyen DP, Salazar-Bravo J, et al. Receptor determinants of zoonotic transmission of New World hemorrhagic fever arenaviruses. Proceedings of the National Academy of Sciences of the United States of America. 2008 Feb 19;105(7):2664-9.

Ref 45.	Cuevas CD, Lavanya M, Wang E, Ross SR. Junin virus infects mouse cells and induces innate immune responses. Journal of virology. 2011 Nov;85(21):11058-68.

Ref 46.	Hickerson BT, Sefing EJ, Bailey KW, Van Wettere AJ, Penichet ML, Gowen BB. Type I interferon underlies severe disease associated with Junín virus infection in mice. eLife. 2020 May 26;9.

Ref 47.	Droniou-Bonzom ME, Reignier T, Oldenburg JE, Cox AU, Exline CM, Rathbun JY, et al. Substitutions in the glycoprotein (GP) of the Candid#1 vaccine strain of Junin virus increase dependence on human transferrin receptor 1 for entry and destabilize the metastable conformation of GP. Journal of virology. 2011 Dec;85(24):13457-62.

Editor’s comments.

There is one small additional change that I think is needed. You responded to one of the original comments regarding the use of mouse TfR1 as a receptor "Radoshitzky reported that MACV does not use murine TfR1 as a receptor as efficiently as human TfR1 in their experiments using pseudotype virus (44)."

I think it's pretty clear that MACV (and JUNV) does not use mouse TfR1 as a receptor, not just that it is inefficient; this has been shown with replication-competent viruses as well as pseudoviruses (please cite references PMCID: PMC7297529 and PMCID: PMC3233171). I would rephrase this section to say "MACV doesn't not use murine TfR1 as a receptor. Further studies are need to clarify the contribution of GPC glycans...."

Once you modify the manuscript accordingly, we would be happy to accept it.
